# Toward a Brain-Computer Interface- and Internet of Things-Based Smart Ward Collaborative System Using Hybrid Signals

**DOI:** 10.1155/2022/6894392

**Published:** 2022-04-18

**Authors:** Xugang Cai, Jiahui Pan

**Affiliations:** ^1^School of Software, South China Normal University, Guangzhou 510631, China; ^2^Pazhou Lab, Guangzhou 510330, China

## Abstract

This study proposes a brain-computer interface (BCI)- and Internet of Things (IoT)-based smart ward collaborative system using hybrid signals. The system is divided into hybrid asynchronous electroencephalography (EEG)-, electrooculography (EOG)- and gyro-based BCI control system and an IoT monitoring and management system. The hybrid BCI control system proposes a GUI paradigm with cursor movement. The user uses the gyro to control the cursor area selection and uses blink-related EOG to control the cursor click. Meanwhile, the attention-related EEG signals are classified based on a support-vector machine (SVM) to make the final judgment. The judgment of the cursor area and the judgment of the attention state are reduced, thereby reducing the false operation rate in the hybrid BCI system. The accuracy in the hybrid BCI control system was 96.65 ± 1.44%, and the false operation rate and command response time were 0.89 ± 0.42 events/min and 2.65 ± 0.48 s, respectively. These results show the application potential of the hybrid BCI control system in daily tasks. In addition, we develop an architecture to connect intelligent things in a smart ward based on narrowband Internet of Things (NB-IoT) technology. The results demonstrate that our system provides superior communication transmission quality.

## 1. Introduction

Traditional artificial care is no longer suitable for the ward environment, where the aging general population is leading to an increase in the patient population. Technologies such as brain-computer interfaces (BCIs) and the wearable Internet of Things (WIoT) are fundamentally changing the current state of medical care and interaction [1–3]. They offer hope for improved interaction, relief of medical pressure, postoperative recovery, and the ability to better address disability and chronic health problems.

Patient-centered care (PCC), which is one of the most important bases for judging the quality of care in a ward [4], is related to the autonomous interaction between the patient and the outside world [4–6]. At present, different hospitals have adopted different ways to control operations in their wards. Due to the lack of convenience, traditional manual control is gradually being replaced by a control mode based on human-computer interaction [7]. Traditional manual help or button control is not intelligent and relies on a singular control mode. Ultimately, this control approach is not suitable for patients with spinal cord injuries, amyotrophic lateral sclerosis, or other diseases that cause full or partial loss of control of their limbs.

With such a wearable device, the electrode signals are collected and analyzed on the cerebral cortex, and the control is realized in combination with computer software and hardware. Currently, motor-impaired individuals and speech-impaired individuals can control a virtual computer cursor [8] and computer keyboard [9, 10] to browse the web by means of noninvasive BCIs. Due to the inconvenience for patients with quadriplegia, many scholars have proposed BCIs based on steady-state visual evoked potential (SSEVP) and motor imagery (MI) to support wheelchair mobility to assist patients in walking [11], to control robotic arms instead of manual grasping [12–15], to control a robotic arm in place of an artificial arm [16], and to enable interaction by means of various other assistive devices [17, 18]. Although single-mode BCIs have achieved some promising results in the abovementioned studies, hybrid BCIs based on multimodal signals and shared control systems can offer improved accuracy and interaction efficiency [19]. Song et al. improved the accuracy of vigilance estimation based on EEG and EOG multimodality [20, 21]. Li et al. presented a hybrid BCI system that combines P300, SSEVP, and MI signals to control a wheelchair [22], which compensates for the limited number of commands available in a single-mode MI BCI (typically 2 or 3) but also leads to a longer response time and a higher user workload. Electrooculography (EOG) has also been used to implement device control based on eye movements [1, 23, 24], such as gazing, blinking, winking, and frowning. Huang et al. proposed a novel hybrid BCI control system based on electroencephalography (EEG) and EOG [12]. In this system, the user performs left/right hand MI to turn a wheelchair left and right and controls a robotic arm by blinking and raising his or her eyebrows. In another study [25], EEG and EOG were used in two separate modes. The EOG mode was used to detect eye movements, including blinking, frowning, winking, and gazing. In EEG mode, multicomponent event-related potentials (ERPs) were adopted to judge the user's visual focus. Although the results achieved with hybrid EOG BCIs suggest that such a hybrid interface is promising for BCI-related applications, the following challenges remain: (I) there is a need to reduce false control commands triggered in the nonblinking state. (II) The accuracy with which blinking and other types of eye movements can be distinguished needs to be further improved.

In addition to PCC, safe, effective, timely, efficient, and equitable care is also a determinant of medical quality [4]. Therefore, monitoring and management are important to ensure safety and provide timely services. The traditional artificial method of vital sign monitoring is time-consuming and burdensome and is affected by the level of experience of nurses [26]. WIoT technology provides a new paradigm in which wearable information-sensing equipment can be connected via the internet [27]. Huang et al. proposed the concept of the internet of health things, in which patient data are aggregated in hospital wards to improve ward efficiency, provide protection, support the optimization of resources, and minimize patient health deterioration [23]. Patient vital sign data can also be used for early warning to reduce morbidity and mortality. Each patient's vital sign data are transmitted to the cloud via WIoT communication [28] and used as input to current artificial intelligence (AI) tools to perform model calculations to infer the possibility of future diseases [29], provide early warning, reduce patient morbidity and mortality, assist in medical care through monitoring, and optimize hospital resources. For the prevention and treatment of infection, WIoT monitoring can be fully automated and more accurate than humans. Shi et al. proposed an intelligent reminder and administration system for hand hygiene (IRAHHS) based on radio frequency identification technology and intelligent analysis technology, which can record and remind medical staff who come in contact with sources of contamination [30]. Current forms of IoT and AI technologies can help the majority of patients; however, some patients with physical disabilities are unable to complete the requirements for interacting with these systems. Meanwhile, due to the relevance of mood and depression [31, 32], modern wards should also focus on the patient's mood and adopt a corresponding regulatory mechanism.

To address these issues, we propose a BCI- and IoT-based smart ward collaborative system using hybrid signals. In particular, our system relies on a GUI paradigm and BCI processing. The GUI is composed of a cursor and multiple buttons. The user uses the gyroscope to control the selection of the cursor area and uses the blinking-related EOG to control the cursor click. The user receives prompts for eye movements (blinks) and simultaneously records them. The user's EEG and EOG signals are identified, and the synchronous selection and transmission of control commands are performed. To verify the usability and reliability of the proposed BCI system for users, we conducted two online experiments with 9 healthy subjects in a smart ward sandbox scenario. The results of these two experiments show that the proposed hybrid BCI system oriented toward the smart ward control paradigm based on EEG + EOG can provide accurate, quick, and efficient control capabilities. The addition of the EEG mode can effectively reduce the number of false operations, indicating that it has the potential to improve the self-nursing ability of patients. In addition, wearable devices and camera equipment can collect physiological signals and other monitoring signals for patients to realize passive control and management through comprehensive assessment, optimize medical resources, and promote a safe medical system.

## 2. Methods

### 2.1. Data Acquisition

**Figure 1:**
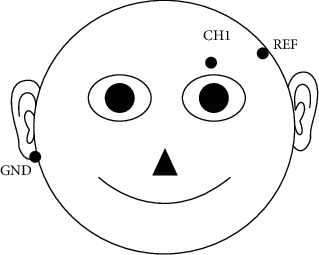
Electrode placement. CH1 (FP1): EEG and EOG electrodes; REF (F7): reference electrode; and GND (A2): ground electrode.

In this study, a customized EEG acquisition device with a sampling rate of 125 Hz was used to collect and amplify raw EEG, EOG, and gyro data. The device mounts three dry electrodes. As shown in Figure 1, electrodes placed on the forehead (F7) and mastoid (A2) on the left side were used as the reference and ground electrodes, respectively. The device extracts the EEG signals and EOG signals from the prefrontal left position (FP1) of the scalp based on the standard positions in the 10–20 system. Three dry electrode sensors were attached to the skin, with the impedance between each of the three electrodes and the skin being lower than 5 kΩ, and the device had 50 Hz power frequency filtering. Meanwhile, multimodal signals used for management and early warning, such as global positioning system (GPS) data, physiological data, environmental monitoring data, and visual data, were obtained through a B2315 wearable wristband device from Oviphone Technology Limited, an environmental monitor, and cameras (more than 8 million pixels).

### 2.2. Control Architecture and GUI

**Figure 2:**
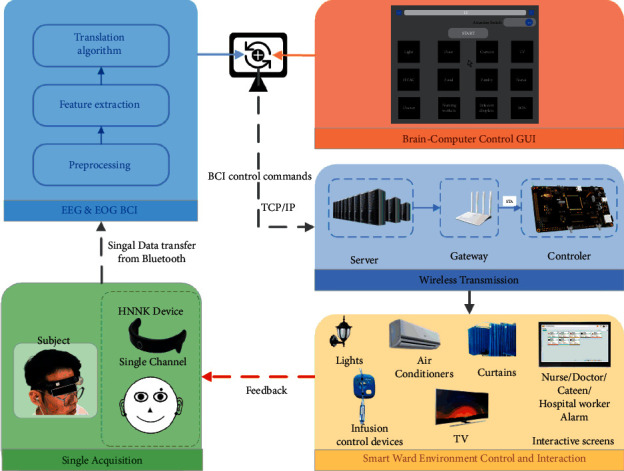
System architecture of the proposed hybrid BCI-controlled smart ward environment.

As shown in Figure 2, the proposed hybrid BCI-controlled smart ward collaborative system mainly consists of a signal acquisition subsystem, an EEG + EOG BCI subsystem, a wireless transmission subsystem, and a smart ward subsystem. The EEG signal is acquired by a customized EEG acquisition device and is then transmitted to a PC, where the data processing for the EEG + EOG BCI subsystem is performed. The EEG + EOG BCI subsystem executes data preprocessing, feature extraction, and a transformation algorithm, and in this subsystem, we include a BCI control GUI to issue EEG control commands. The wireless transmission subsystem transmits the EEG + EOG BCI control commands to the smart ward control subsystem through the TCP/IP protocol. The smart ward subsystem then executes control and interactive operations in accordance with the received EEG + EOG BCI control commands in the smart ward environment.

**Figure 3:**
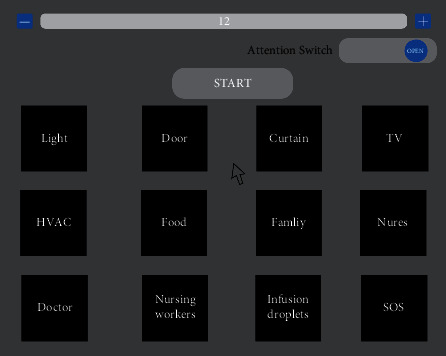
This GUI consists of two layers on a single page. (a) Mode selection layer: 2 command number adjustment buttons, “
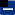
” and “
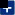
”; an attention switch button, “
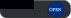
”; and a BCI activation/deactivation button, “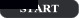.” (b) Command control layer: 12 control buttons for the ward environment.

As illustrated in Figure 3, a two-layer single-page GUI structure is used for the control of the smart ward environment. The two layers are (a) a mode selection layer and (b) a command control layer. The mode selection layer consists of four buttons. “
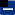
” and “
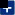
” are command number adjustment buttons; “
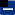
” reduces the number of commands; “
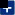
” increases the number of commands; and “
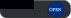
” is a button for attention switching. The command control layer is adjusted with the “
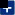
” and “
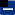
” button commands, displaying between 4 and 12 ward-specific control command buttons. “
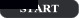
” is the button to activate/deactivate BCI control. After a command is clicked, the next click ends the BCI control task. The GUI structure also includes a mouse cursor with a controllable head. When the system is turned on, the GUI panel is presented, and the user turns his or her head and blinks to select the mode configuration (default: 12 ward-specific control commands and attention close). The user turns his or her head and blinks to select a ward-specific control command. The cursor's movement in the two-dimensional plane is controlled by the gyro, and a blinking action realizes a cursor click. In addition, when a control command is successfully implemented, the command button will be highlighted for 500 ms to remind the user of which command has been selected.

In our system, button selection is used to execute the control command task. To prevent natural blinking in the idle state from causing mistaken selection, the user should close the control GUI panel to reduce the incidence of false commands issued in the idle state.

### 2.3. Detection Algorithm

#### 2.3.1. Attention Detection

Attention detection includes three processes: signal preprocessing, feature extraction, and classification and recognition.

*(1) Signal Preprocessing*. In this study, a moving time window is used to divide the EEG signal. The length of the time window is 5 s, and the length of the EEG signal is 625. The EEG signal is debaselined to reduce drift and DC interference. Then, low-pass filtering and high-pass filtering are performed. In particular, a third-order Butterworth filter with a cutoff frequency of 60 Hz is adopted for low-pass filtering, and a third-order Butterworth filter with a cutoff frequency of 0.1 Hz is adopted for high-pass filtering. After filtering, the waves in various frequency wavebands are obtained, including *δ* (1∼3 Hz), *θ* (4∼7 Hz), *α* (8∼13 Hz), *β* (14∼30 Hz), and *γ* (31∼48 Hz).

*(2) Feature Extraction*. Spontaneous EEG signals are divided into five types according to their frequency wavebands: *δ*, *θ*, *α*, *β,* and *γ* waves, among which *α*, *β*, *θ*, and *γ* signals are related to attention [33, 34]. In this study, the Welch algorithm is used to estimate the power spectral density. First, the finite-length observation sequence *x*(*n*)(*n*=1,2, ..., 625) is divided into four segments after preprocessing, where the length of each segment is 250 and the number of overlapping data points between adjacent segments is 125. Then, each segment is processed with a Hamming window and subjected to a fast Fourier transform (FFT) at the same time. Finally, the average is taken to obtain an estimate of the power spectrum of the signal. The resolution of the estimated power spectrum is 1 Hz. The calculation formula is as follows:

(1) $$\begin{array}{c} \mathrm{PS}D_{i}\left(\mathrm{freq}\right)=\frac{1}{\mathrm{MU}}{\left|\sum_{n=0}^{M-1}x_{i}\left(n\right)w\left(n\right)e^{-j2\pi freq_{i}}\right|}^{2}, \end{array}$$

where *i* represents the EEG signal of the *i*-th segment, *M* is the segment length, *w*(*n*) is the Hamming window function, and *U* is the calculated power of the window function. The calculation formula is as follows:

(2) $$\begin{array}{c} U=\frac{1}{M}\sum_{n=0}^{M-1}w^{2}\left(n\right),\\ P_{\text{freq}}=\frac{1}{4}\sum_{i=0}^{3}PSD_{i}\left(\text{freq}\right). \end{array}$$

The summation of the energy values produces five features, in accordance with the waveband distribution of the EEG signal. Let *P*_freq_ denote the energy value corresponding to the frequency freq; then, the features extracted for attention recognition can be defined as follows:

(3) $$\begin{array}{c} E_{\delta }=\sum_{\text{freq}=1}^{3}P_{\text{freq}},\\ E_{\theta }=\sum_{\text{freq}=4}^{7}P_{\text{freq}},\\ E_{\alpha }=\sum_{\text{freq}=8}^{13}P_{\text{freq}},\\ E_{\beta }=\sum_{\text{freq}=14}^{30}P_{\text{freq}},\\ E_{\gamma }=\sum_{\text{freq}=31}^{48}P_{\text{freq}}. \end{array}$$

In addition, the ratio between *α* and *β* activities can be used as a feature for assessing the level of mental attentiveness [33]. In this study, the following feature value is calculated using this principle:

(4) $$\begin{array}{c} R=\frac{E_{\alpha }}{E_{\beta }}, \end{array}$$

where *R* is also a feature used to determine whether the user is attentive. Therefore, in this study, a total of six features are extracted as the basis for classification.

*(3) Classification and Recognition*. In this study, a support-vector machine (SVM) classifier with excellent classification performance is employed to separate EEG signals. Attention samples (attention and nonattention) for training and testing were collected based on the test of variables of attention (T.O.V.A.)[35], and a linear function was adopted as the kernel function of the SVM model. Fivefold cross validation was performed to find the optimal parameters of the model, and finally, the SVM model was trained based on the optimal parameters.

#### 2.3.2. Blink Detection

*(1) Signal Preprocessing*. In this study, button selection is achieved by means of blinking-related vertical EOG signals. In particular, the 600 ms EOG signal is intercepted after finding the starting point of blinking asynchronously based on the moving average method, and the EOG signal is filtered through a 0.1–10 Hz bandpass filter to remove high-frequency components.

**Figure 4:**
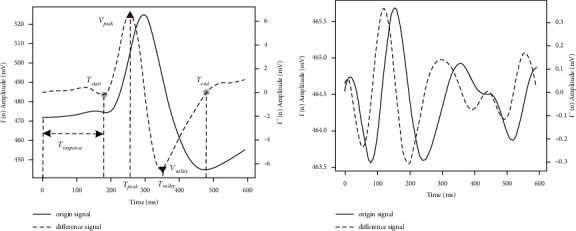
EOG features for blink detection: (a) blinking EOG waveform and (b) nonblinking EOG waveform. Solid curve: original EOG waveform; dashed curve: first-order derivative of the EOG signal waveform; upper and lower triangle marks: peak amplitude and valley amplitude; and black solid points: starting point and ending point.

*(2) Feature Extraction*. The first-order derivative operation is applied to the preprocessed signal to obtain its feature vector. In this manner, a corresponding feature vector is extracted immediately after each button flashes. Previous studies have shown that different kinds of eye movements, such as gazing, winking, blinking, and frowning, have different amplitudes and durations. Moreover, the peak occurs before the corresponding valley. Therefore, as shown in Figure 4, for a blinking EOG waveform (a) and a nonblinking EOG waveform (b), we extract the peak amplitude (*V*_peak_), valley amplitude (*V*_valley_), peak time (*T*_peak_), valley time (*T*_valley_), reaction time *T*_reaponse_, and duration *T*_*d*_=*T*_end_ − *T*_start_ features from these waveforms for blink detection.

*(3) Waveform Detection*. In this study, blink detection is performed for each button flash based on threshold conditions. First, the following equations are used to calculate the duration and energy of each feature vector:

(5) $$\begin{array}{c} d=T_{\text{end}}-T_{\text{start}},\\ e={\sum_{t=T_{\text{peak}}}^{T_{\text{valley}}}\left(f^{\prime }\left(n\right)\right)}^{2}. \end{array}$$

Subsequently, these special vector diagnostic features *e* and *d* are compared against certain thresholds, which are chosen based on experience, to determine whether a blink is detected. For the successful detection of blinking, the following inequalities must be satisfied:

(6) $$\begin{array}{c} e>E,\\ T_{\text{peak}}>T_{\text{valley}},\\ D_{\min}\leq d\leq D_{\max}, \end{array}$$

where *d* and *e* represent the blink speed and strength, respectively; *E* is the minimum energy threshold; *D*_min_ is the minimum duration; and *D*_max_ is the maximum duration. If a feature vector meets the above requirements, a blinking waveform is detected corresponding to the associated button, and the result is 1; otherwise, the result is 0, and a blinking waveform corresponding to the associated button is not detected.

#### 2.3.3. Cursor Area Selection

In this study, the cursor movement on the GUI is controlled by the rotation of the head ring. In particular, the cursor posture is analyzed based on the quaternion complementary filtering algorithm.

*(1) Signal Preprocessing*. Gyro data and acceleration data are obtained from an MPU-6050 motion sensing module. The gyro data are calibrated with zero drift, and the acceleration data are filtered using an extreme sliding window.

*(2) Attitude Analysis and Transformation*. Subsequently, the gravity component is obtained based on the quaternion, and the error between the measured and estimated gravity vectors is calculated as the difference between them. The obtained error is used to correct the gyro measurement value, and the corrected gyro value is updated with the quaternion. Finally, the quaternion is standardized and transformed into Euler angles.

#### 2.3.4. Multimodal Decision-Making

**Figure 5:**
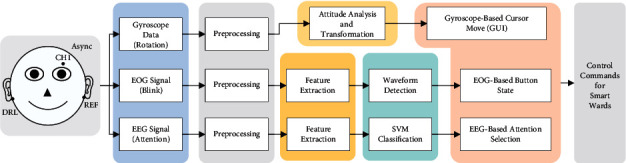
Flowchart of the entire system. The EOG signal, EEG signal, and gyro data are acquired and simultaneously fed into three different data processing procedures. In particular, in asynchronous mode, the cursor movement is implemented based on the gyro data and button selection is implemented based on the EOG and EEG signals.

As shown in Figure 5, button selection is performed through multimodal fusion control. In particular, the EEG and EOG signals will be recorded and stored in real time at a frequency of 125 Hz. The cursor is allowed to move, and four steps need to be performed for the user to issue a control command:

  Step 1: the gyro transmits data for posture analysis to the PC in real time, and these data are used to control the movement of the cursor in the two-dimensional plane of the GUI on the PC. The user uses the gyroscope to control the cursor to move to the preselected button area and uses the blinking-related EOG to control the cursor click.
  Step 2: the EEG and EOG signals will be recorded and stored in real time at a frequency of 125 Hz. The system uses the moving average method to identify the starting point of the blinking EOG waveform in real time. At the same time, the 5 EEG signals (625 sampling points) before the starting point, the cursor area coordinates at the starting point, and the EOG signal (75 sampling points) 600 ms after the starting point are extracted.
  Step 3: the EOG signal and the EEG signal are synchronously processed by the algorithm, the EOG signal is based on waveform detection for blink detection, and the EEG signal is based on SVM for attention state classification.
  Step 4: finally, the recognition and classification results meet the threshold requirements, and the button command covering the area selected by the cursor is the final control command.

### 2.4. Monitoring of Multiple Biological Signals

A multimodal smart ward collaborative system that integrates multiple signals can simplify and integrate the ward management process, including vital sign monitoring, environmental monitoring, and automatic control.

**Figure 6:**
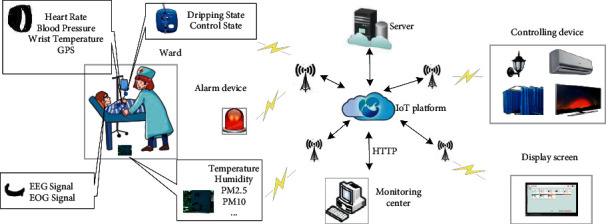
Monitoring and management system architecture.

In particular, as shown in Figure 6, the monitoring and management system can be divided into three layers. The first layer is the perception layer, which is composed of various sensors. It is responsible for collecting data on the internal electrical state of the ward, environmental monitoring data, and physiological and positioning data for the patient in the ward. The collected data are passed through a microcontrol unit (MCU) for code integration and transferred to a narrowband Internet of Things (NB-IoT) module. The second layer is the transport layer. The NB-IoT chip automatically encapsulates the payload into a constrained application protocol (CoAP) message and simultaneously transmits the data to nearby communication base stations. CoAP is designed to solve the problem of limited device resources in the IoT context, where the traditional hypertext transfer protocol (HTTP) is often not applicable. The third layer is the platform application layer. The cloud server is authorized to share and store the data collected by the sensors from the patient's body. At the monitoring center, the physiological and medical data are displayed on the patient monitor in real time, and the positioning and environmental data are displayed on the safety monitor in real time. Meanwhile, cloud computing and network technology are closely integrated to make decisions, analyze the data, and monitor the ward for the patient's safety state in real time. In addition, commands can be sent to the lower-level control module to control the electrical equipment to realize automatic control.

## 3. Experiments and Results

### 3.1. Experimental Process

In this study, two types of online experiments were performed to verify the proposed system. The first was asynchronous online experiments, using an IoT sandbox as the experimental control equipment. The second experiment was an online monitoring and management experiment. The details of the experiments are introduced in this section.

Nine healthy volunteers (numbered *S*1∼*S*9) aged between 21 and 26 years participated in this study. Among them, three (*S*4, *S*7, and *S*8) were women, and the others were men. It should be noted that *S*1∼*S*3 had experimental experience with BCIs, while the others did not. All subjects reported normal vision or corrected-to-normal vision. Eight subjects, *S*1∼*S*8, participated in the online asynchronous experiment, and *S*14 participated in the IoT experiment.

**Figure 7:**
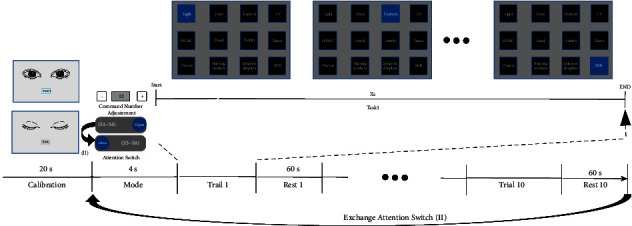
The online asynchronous experiment. In the calibration phase, 10 blink prompts were presented at intervals of 20 s. In the mode selection stage, 4 s of time was provided to complete command number adjustment and attention switch selection. In the online experimental phase, there were 10 subtrials; in each subtrial, 6 commands were required to be selected, and after each subtrial, there was a 60 s rest period before the next subtrial began. In the end, the subject switched the attention switch to perform another round.

Figure 7 illustrates the detailed timelines of a single run of asynchronous online Experiment 1. The performance indices used in this study are listed as follows:

- *Accuracy (ACC)*: the probability of correctly selecting a button
- *Response time (RT)*: the time required to generate a command
- *Information transfer rate (ITR)* [33]: the number of bits of information transferred per minute
- *False operation rate (FOR)*: the number of false operations occurring per minute in the idle state

**Experiment 1 .**

To test interaction and control by moving the cursor and using the visual command button interface (asynchronous online implementation), the following steps were completed:

1. Calibration: each subject performed 10 blinking actions in accordance with the presented blink command prompts. The interval between consecutive blinks was 2 s, and the total time was 20 s.
2. Start task: twelve control command buttons are adjusted by means of “plus/minus” buttons. Then, attention detection was turned on for 4 subjects (*S*1∼*S*4) and turned off for 4 subjects (*S*5∼*S*8). After the mode configuration was completed, the subjects selected the “start” button to start the BCI control task. The total time was 4 s.
3. Each subject selected the target button in accordance with the presented prompts and completed 6 operations: turning on the lights, opening the curtains, turning on the TV, calling the doctor, turning off the infusion set, and making an emergency call. During this period, incorrect control commands could appear, and the subjects needed to correct them until the correct command was obtained.
4. End task: the subjects returned to the task start GUI.
5. Steps (2) to (4) were repeated 10 times, with a rest time of 1 minute each time.
6. Finally, subjects *S*1∼*S*4 turned off attention detection, subjects *S*5∼*S*8 turned on attention detection, and all subjects then repeated steps (2) to (5).

**Experiment 2 .**

This experiment was divided into three subexperiments to verify the accuracy and reliability of the system:

  Subexperiment A: an environmental monitoring module was placed in a representative simulated ward sandbox for 12 hours of continuous monitoring. Meanwhile, the monitored values reported by an RS-MG111-N01-1 sensor (Shandong Renke Control Technology Co., Ltd.) were recorded as the control standard every 4 hours. This sensor has high measurement accuracy, with a temperature accuracy of ±0.3°C, a humidity accuracy of ±3% RH, formaldehyde (HCHO) concentration accuracy of ±2.5%, and a PM2.5 accuracy of ±5%.
  Subexperiment B: subject *S*14 was required to wear the bracelet used in our system and an Apple Watch Series *S*6 at the same time for a 40-minute heart rate experiment. The experiment was divided into 4 activity sets: sitting, walking, running, and walking. Each set lasted 10 minutes, and heart rate data were collected from the subject at intervals of one minute during that time.
  Subexperiment C: to verify the stability of system data transmission, first, a terminal node cyclically sent data packets (16 bytes) to the cloud server. The interval time was 1 s, and the transmission test was divided into 5 groups, where the total number of data packets sent in each group was different. Subsequently, another terminal node was introduced as an interfering node, and a comparative experiment was performed with the same total number of data packets.

### 3.2. Experimental Results

The results of Experiment 1 are shown in Tables 1 and 2. All indicators in this experiment were averaged for each subject. In asynchronous mode, 8 subjects completed a set of control experiments with attention detection turned on and with attention detection turned off. In this mode, all subjects made button selections as quickly as possible. If a false choice occurred in the middle of the selection process, the subject needed to correct it and record the false event until the correct order was issued. In this mode, there is no need to wait for the flashing time of the preselect button in synchronous EOG mode [23]. Consequently, the time to generate a command was greatly reduced; in particular, the RT was 2.87 ± 0.49 s (attention closed) and 2.65 ± 0.48 s (attention open). When attention detection was turned off, the overall ACC, ITR, and FOR of the system were 95.54 ± 1.28%, 47.43 ± 7.62 bits/min, and 1.10 ± 0.32 bits/min, respectively; when attention detection was turned on, the values of these indices were 96.65 ± 1.44%, 53.42 ± 8.44 bits/min, and 0.89 ± 0.42 bits/min, respectively.

The online asynchronous experiment fairly comprehensively demonstrates the performance of the system and shows that the performance of multimodal fusion control is satisfactory.

**Figure 8:**
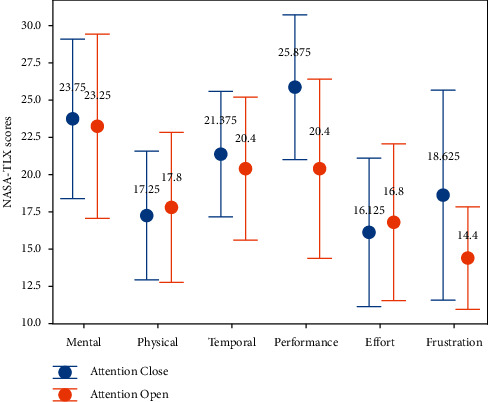
Subjective workload assessment results for Experiment 1. The means and standard deviations of the NASA-TLX scores are represented by circles and error bars, respectively.

To measure the workload involved in the proposed hybrid BCI system, once Experiment 1 was completed, the 8 subjects were asked to independently complete a workload questionnaire following the NASA Task Load Index (TLX) method of Hart and Staveland [36]. This questionnaire evaluates workload in terms of six aspects: mental demand, physical demand, temporal demand, overall performance, effort, and frustration level. As shown in Figure 8, the average scores for all 6 factors in the two modes remained below 26. The overall average scores for the two states in the experiment were roughly the same. In particular, the overall average scores for the two states in Experiment 1 were roughly the same, which shows that reducing error rates can improve individual performance satisfaction and reduce frustration. In general, our proposed hybrid BCI based on EEG, EOG, and gyro signals was acceptable.

**Figure 9:**
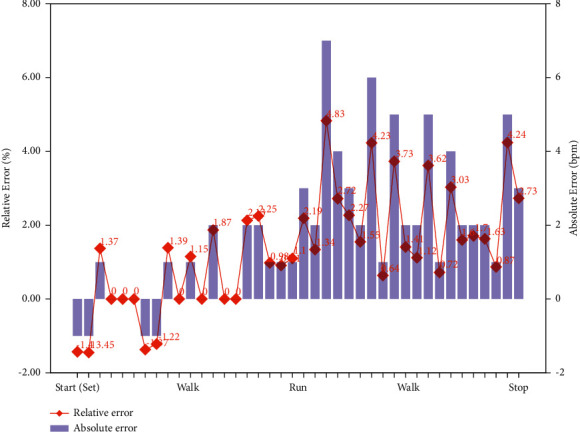
Absolute and relative error results for the measured and standard heart rate values.

From the results of Experiment 2 reported in Table 3, it can be concluded that the cloud platform could accurately and reliably display the monitoring data. Our system can obtain real-time information on environmental parameters such as temperature, humidity, HCHO, and PM2.5. In particular, the temperature measurement accuracy was maintained within ±1%, the humidity accuracy was maintained within ±2%, the HCHO accuracy was maintained within 3%, and the PM2.5 accuracy was maintained within ±5%. Thus, compared with the standard values, the relative errors of the measured parameters were very small. Similarly, as shown in Figure 9, our system could accurately and reliably monitor the user's heart rate data. Compared with the standard values, the maximum relative error during running was 4.83%, and the absolute error was 7 bpm. In addition, we divide the data into 5 sets to complete the communication experiment of 600 to 6,000 data packets. The 5 sets of transmission experiments proved the reliability of network communication. As shown in Table 4, in the absence of an interfering node, the packet loss rate and bit error rate of network communication over 10 minutes were 0.1% and 0%, respectively. Under interference from another node, the packet loss rate and bit error rate were 0.2% and 0.8%, respectively.

## 4. Discussion

In this work, we presented a BCI- and IoT-based smart ward collaborative system using hybrid signals. The experimental results of eight subjects showed that the average accuracy of the hybrid BCI system was 95.54 ± 1.28% and 96.65 ± 1.44%, and the ITR was 47.43 ± 7.62% and 53.42 ± 8.44%, respectively. Attention-related EEG analysis effectively reduces the FOR of the BCI system. The use of gyro data endows the asynchronous BCI system with a higher RT and lower FOR. In addition, the IoT monitoring and management system is developed based on NB-IoT technology. The experimental results show that the packet loss rate and bit error rate of network communication are both lower than 1%, thus proving the reliability of our system.

In a traditional ward environment, users perform control functions through manual operations; however, the size and performance of the manual control equipment limit the number of commands that can be issued. In this study, we propose a hybrid BCI system based on EEG, EOG, and gyro signals, which provides a novel control method that allows users to overcome certain physical limitations. Generally, an EOG signal exhibits obvious patterns that are relatively simple to detect, and consequently, a BCI system based on EOG imposes a low workload and simple operation requirements [37]. Conversely, BCI systems based on ERPs (P300 and SSEVP) [13, 16, 22]and MI-based BCI systems often have high workloads and require a long training time. In addition, an ERP-based BCI typically has a low ITR or high FOR in either the control or idle state, while an MI BCI commonly has a long RT. However, the number of possible commands in an EOG-based BCI system is limited by the different types of eye movements that can be recognized. For this reason, we propose a hybrid EEG + EOG method for button selection. We propose an EOG cursor interaction GUI. The cursor moves to the button area for blinking and clicks, and the richness of control instructions is improved. Furthermore, RT is another important indicator used to assess an asynchronous system. In this study, compared with other synchronous button blinking guidance methods [12, 23, 24], the gyroscope cursor moving method does not need to wait for the guidance blinking time and provides faster instruction completion speed (average RT: 2.87 ± 0.49/2.65 ± 0.48).

A major challenge in an EOG-based BCI system is to avoid false operations in the idle state when the user involuntarily blinks, which may trigger a control signal. Therefore, a single-mode system based on blinking alone will tend to have a high FOR. In recent years, to ensure the ability to accurately distinguish between intentional blinking and the idle state, many scholars have attempted to develop EOG-based BCI systems using multiple blinks and different eye movement patterns, such as two blinks [25, 38], three blinks [25], blinking a single eye [39], and saccades [24, 40]. In our study, the user confirms that the final command needs to meet two conditions in addition to recognizing the blink action: (1) the user's cursor area is a button area instead of a nonbutton area, and (2) the user's EEG attention state classification result before blinking action needs to satisfy the nonidle condition. The two conditions effectively reduce the natural blink false operations in the idle state and improve the system robustness. In particular, the system determines the blinking action based on waveform detection. At the same time, cursor area determination and attention state SVM classification are simultaneously performed with waveform detection. If the three conditions are satisfied to execute the final command, if one of the three conditions is not satisfied, the command is determined to be invalid, and no operation is performed. As shown in Tables 1 and 2, the introduction of this attention state method significantly reduces the FOR. Meanwhile, our system analyzes the cursor position based on the quaternion complementary filtering algorithm. In contrast to the method based on MI and EOG presented by He et al. [41], our asynchronous system does not require training and is easier to operate. In addition, fixation duration, saccade frequency, and duration affect subjects' mental load and reduce task efficiency [42]. Next, we will improve attention based on finding a reasonable fixation duration and task duration.

Another challenge in an EOG-based BCI system is to increase the ITR. The core requirement is to maintain the correct rate while decreasing the RT. For this purpose, it is crucial to accurately identify the blinking action of the EOG. In particular, active blinking yields a stronger EOG signal than autonomous blinking or no blinking; the energy and duration are both greater [43]. The asynchronous BCI system has no synchronous blinking induction and can accurately find the onset point of the EOG signal suspected of blinking. Based on the moving average method, the point of signal transition is found from the comparison between the actual collected value and the predicted value, and then, the 600 ms EOG signal is intercepted for waveform detection. Compared with traditional peak and trough detection [37], the EOG signal has more prominent energy characteristics after first-order difference processing, and the test accuracy is 95.54 ± 1.28/96.65 ± 1.44 in 8 subjects.  Table 5 compares the results of our BCI control system with those of several existing BCI control systems. Our system shows advantages in terms of the ACC and FOR, and the ITR is also good, although lower than that in [44] in synchronous mode, because the button flashing time interval is shorter in the latter system, thereby shortening the RT; however, this will also affect the classification accuracy.

Furthermore, accurate and reliable data collection to facilitate monitoring is a major challenge in the establishment of smart wards. The proposed system uses NB-IoT technology to integrate environmental and physiological data collected in the ward to form a safe and effective smart ward monitoring and management platform. In particular, an NB-IoT system can be established using an existing network architecture (such as long-term evolution (LTE) or global system for mobile communications (GSM)) to achieve low-cost and rapid deployment. This technology also meets the needs of massive machine-type communication (mMTC) scenarios in fifth-generation (5G) networking [48]. At the same time, NB-IoT offers coverage enhancements and greater delay tolerance while featuring low power consumption. For physiological information collection, a convenient wearable device is adopted for daily use. The built-in high-precision sensor ensures that the data are accurate and reliable. The heart rate monitoring accuracy is guaranteed to remain within 5%, and the average relative error in the sitting state is 0.82%. In addition, the introduction of GPS and environmental monitoring data into the system can comprehensively ensure user safety. As seen from the results of an information transmission test, the packet loss rate and bit error rate of network communication are less than 1% regardless of the presence of interfering nodes. The experimental findings show that the system stably runs and achieves the expected results.

## 5. Conclusions

This study proposes a BCI- and IoT-based smart ward collaborative system using hybrid signals. The system is divided into a hybrid EEG-, EOG-, and gyro-based BCI system and an IoT monitoring and management system. The experimental results of 8 subjects showed that the accuracy and ITR of the hybrid BCI were 96.65 ± 1.44% and 53.42 ± 8.44, respectively. Attention-related EEG analysis effectively reduces the FOR of the BCI system. Meanwhile, the use of gyro data endows the asynchronous BCI system with a higher RT and lower FOR. In addition, to ensure user safety, the IoT monitoring and management system is developed based on NB-IoT technology. The experimental results show that both environmental data and physiological data are accurately monitored. The packet loss rate and bit error rate of network communication are both lower than 1%. Our system provides a novel control method for disability and chronic patients, which effectively improves accuracy and reduces FOR. At the same time, it provides a safe and reliable monitoring system for medical care and realizes PCC. In future work, we will continue to improve the hybrid BCI system by further optimizing the eye-tracking method and integrating IoT resources to make the proposed smart ward collaborative system more user friendly for disabled people.

## Figures and Tables

**Table 1:** Results for the subjects in Experiment 1 (attention closed).

Sub.	Attention state	ITR (bits/min)	FOR (events/min)	ACC (%)	RT (s)
*S*1	Closed	48.59	1.03	95.71	2.75
*S*2	Closed	34.49	1.01	94.29	3.72
*S*3	Closed	47.76	1.39	94.64	2.71
*S*4	Closed	39.37	0.55	97.14	3.55
*S*5	Closed	46.19	1.34	94.64	2.80
*S*6	Closed	56.05	0.77	97.50	2.52
*S*7	Closed	50.06	1.47	94.29	2.56
*S*8	Closed	56.91	1.21	96.07	2.38
Avg	—	47.43 ± 7.62	1.1 ± 0.32	95.54 ± 1.28	2.87 ± 0.49

**Table 2:** Results for the subjects in Experiment 1 (attention open).

Sub.	Attention state	ITR (bits/min)	FOR (events/min)	ACC (%)	RT (s)
*S*1	Open	55.28	0.77	97.14	2.53
*S*2	Open	37.91	0.81	95.71	3.53
*S*3	Open	60.85	1.30	95.71	2.20
*S*4	Open	45.13	0.30	98.57	3.25
*S*5	Open	61.41	0.85	97.14	2.28
*S*6	Open	60.09	0.40	98.57	2.44
*S*7	Open	56.69	1.21	95.71	2.36
*S*8	Open	50.00	1.46	94.64	2.59
Avg	—	53.42 ± 8.44	0.89 ± 0.42	96.65 ± 1.44	2.65 ± 0.48

**Table 3:** Comparison of measured and standard monitoring values in the ward environment.

Parameter	Time	Standard values	Measured data by our system	
			Measured values	Relative error (%)
Temperature °C	6:00	27.22	27.02	−0.73%
	10:00	31.95	32.01	0.19%
	14:00	34.56	34.9	0.98%
	18:00	31.22	31.03	−0.61%
Humidity % RH	6:00	65.6	65.63	0.05%
	10:00	60.13	60.13	0.00%
	14:00	58.17	57.08	−1.87%
	18:00	58.98	58.53	−0.76%
HCHO mg/m^3^	6:00	0.0596	0.0588	−1.34%
	10:00	0.0608	0.0612	0.66%
	14:00	0.0644	0.0636	−1.24%
	18:00	0.0629	0.0612	−2.70%
PM2.5 ug/m^3^	6:00	101	104.0000	2.97%
	10:00	110	115.0000	4.55%
	14:00	113	118.0000	4.42%
	18:00	113	117.0000	3.54%

**Table 4:** Results of the network communication test.

Number of sent data packets (pcs)	No interfering node			Interfering node		
	Received data (pcs)	Packet loss rate (%)	Bit error rate (%)	Received data (pcs)	Packet loss rate (%)	Bit error rate (%)
600	600	0	0	600	0	0
1200	1200	0	0	1200	0	0.3
2400	2400	0	0	2396	0.2	0.5
4800	4799	0.1	0	4797	0.1	0.4
6000	5996	0.1	0	5989	0.2	0.8

**Table 5:** Comparison with other BCI systems.

Publication	Mode (s)	ACC (%)	ITR (bit/min)	FOR (event/min)
He and Li [37]	EOG	93.02	45.83	—
Huang et al. [23]	EOG	91.7	48.8	0
Kubacki [15]	EEG + EOG	90.0	—	—
Chen et al. [16]	EEG + CV	94.0	14.21	—
Zhang et al. [45]	EEG	89.9	23.2	—
Zhou et al. [46]	EEG	92.8	≤26.3^*∗*^	≥6.71^*∗*^
Zhang et al. [24]	EOG	93.6	26.8	—
Shao et al. [44]	EEG + EOG	95.42	105.5	0.80
Zhang et al. [47]	EEG	91.7	≤23.8	≥1.75^*∗*^
**Our system**	**EEG** **+** **EOG** **+** **Gyro**	**96.7**	**53.42**	**0.89**

— denotes not reported ^*∗*^The value was calculated from the results reported by the authors.

## Data Availability

All data included in this study are available upon request to the corresponding author.
